# Development and evaluation study of FLY-Kids: a new lifestyle screening tool for young children

**DOI:** 10.1007/s00431-023-05126-6

**Published:** 2023-08-15

**Authors:** Anne Krijger, Lieke Schiphof-Godart, Liset Elstgeest, Caroline van Rossum, Janneke Verkaik-Kloosterman, Elly Steenbergen, Sovianne ter Borg, Caren Lanting, Karen van Drongelen, Ondine Engelse, Angelika Kindermann, Symone Detmar, Carolien Frenkel, Hein Raat, Koen Joosten

**Affiliations:** 1 Department of Pediatrics and Pediatric Surgery, Erasmus MC-Sophia Children’s Hospital, University Medical Center Rotterdam, PO Box 2060, 3000 CB Rotterdam, The Netherlands; 2 Department of Public Health, Erasmus MC, University Medical Center Rotterdam, Rotterdam, The Netherlands; 3 Department of Medical Informatics, University Medical Center Rotterdam, Rotterdam, The Netherlands; 4 Reinier Academy, Reinier de Graaf Hospital, Delft, The Netherlands; 5 National Institute for Public Health and the Environment, Bilthoven, the Netherlands; 6 Netherlands Organisation for Applied Scientific Research TNO, Unit Healthy Living, Child Health Expertise Group, Leiden, The Netherlands; 7 The Netherlands Nutrition Centre, The Hague, The Netherlands; 8 Dutch Knowledge Centre for Youth Health, Utrecht, The Netherlands; 9 Department of Pediatric Gastroenterology, Hepatology and Nutrition, Amsterdam UMC, University of Amsterdam, Emma Children’s Hospital, Amsterdam, The Netherlands; 10 Association of Dutch Infant and Dietetic Foods Industries, The Hague, The Netherlands

**Keywords:** Lifestyle, Toddlers, Screening, Conversation aid

## Abstract

Evaluating, discussing, and advising on young children’s lifestyles may contribute to timely modification of unhealthy behaviour and prevention of adverse health consequences. We aimed to develop and evaluate a new lifestyle screening tool for children aged 1–3 years. The lifestyle screening tool “FLY-Kids” was developed using data from lifestyle behaviour patterns of Dutch toddlers, age-specific lifestyle recommendations, target group analyses, and a Delphi process. Through 10 items, FLY-Kids generates a dashboard with an overview of the child’s lifestyle that can be used as conversation aid. FLY-Kids was completed by parents of children aged 1–3 years attending a regular youth healthcare appointment. Youth healthcare professionals (YHCP) then used the FLY-Kids dashboard to discuss lifestyle with the parents and provided tailored advice. Parents as well as YHCP evaluated the tool after use. Descriptive and correlation statistics were used to determine the usability, feasibility, and preliminary effect of FLY-Kids. Parents (*N* = 201) scored an average of 3.2 (out of 9, SD 1.6) unfavourable lifestyle behaviours in their children, while 3.0% complied with all recommendations. Most unfavourable behaviours were reported in unhealthy food intake and electronic screen time behaviour. Parents and YHCP regarded FLY-Kids as usable and feasible. The number of items identified by FLY-Kids as requiring attention was associated with the number of items discussed during the appointment (*r* = 0.47, *p* < 0.001).

*     Conclusion:* FLY-Kids can be used to identify unhealthy lifestyle behaviour in young children and guide the conversation about lifestyle in preventive healthcare settings. End-users rated FLY-Kids as helpful and user-friendly.

**Table:** 

**What is Known:** *• A healthy lifestyle is important for optimal growth, development and overall health of young children (1-3 years)*. *• Evaluating, discussing and advising on young children’s lifestyles may contribute to timely modification of unhealthy behaviour and prevention of adverse health consequences*.	
**What is New:** *• The new lifestyle screening tool FLY-Kids generates a dashboard with an overview of young children’s lifestyle that can be used as conversation aid between parents and youth healthcare professionals*. *• As parents and youth healthcare professionals rated FLY-Kids as helpful and user-friendly, and the number of items identified by FLY-Kids as requiring attention was associated with the number of items discussed during the appointment, FLY-Kids can be considered guiding the lifestyle discussion in preventive healthcare settings*.

**Supplementary Information:**

The online version contains supplementary material available at 10.1007/s00431-023-05126-6.

## Introduction

Despite the importance of a healthy lifestyle for children’s optimal growth and development, many parents do not comply with lifestyle recommendations for their offspring [1]. Unfavourable lifestyle behaviour, such as inadequate dietary intake, lack of physical activity, high amounts of screen time, and insufficient sleep, has been associated with adverse health outcomes already in early childhood [2–5]. Overweight and obesity are among the most prominent health implications, with a global prevalence of 5.7% in children under the age of five [6]. In addition to the increased risk of certain (chronic) diseases due to being overweight, common consequences of an unhealthy lifestyle in children include tooth decay, myopia, impaired motor skills, and delayed cognitive development [7–9]. Given that lifestyle habits formed during childhood tend to persevere, as does overweight, the early years provide the perfect opportunity for sustained healthy behaviour and its associated health benefits throughout life [10–12].

Since young children (aged 1–3 years) represent a vulnerable group with high potential, promoting a healthy lifestyle in them should be prioritised. To timely tackle unfavourable lifestyle behaviour of young children, a screening tool may be helpful. Such a tool, completed by parents (or caregivers, also referred to as parents in this paper), would allow young children’s lifestyle habits to be mapped quickly and easily. While using a lifestyle screening tool could create awareness among parents, on the one hand, such tools could also offer healthcare professionals prompts to start a conversation about lifestyle with parents. Consequently, suboptimal lifestyle behaviours could be discussed, and tailored advice can be given to support the parents in improving their child’s lifestyle behaviour.

A few lifestyle screening tools exist for community-living children aged 1–3 years. The Toddler Dietary Questionnaire, NutricheQ, and Toddler NutriSTEP are short screening tools that identify nutritional risk [13–15]. The Toddler Dietary Questionnaire addresses the intake of specific food groups [13]. The NutricheQ and Toddler NutriSTEP additionally encompass aspects such as feeding practices and parent feeding styles (NutricheQ) and growth and daily sedentary activity (Toddler NutriSTEP) [14, 15]. Nevertheless, the outcome of these tools is still limited to nutrition. Another concern in the application of lifestyle screening tools in young children is the feedback and support to parents. While completing a screening tool could lead to awareness, a response to the outcome and advice tailored to the family concerned may increase the chance of actual behavioural change [16]. Furthermore, for successful implementation, healthcare professionals have to be guided in discussing screening tool outcomes and be given specific courses of action. Currently, there is no screening tool that covers lifestyle in the broadest sense of the term with specific action protocols that can be used in preventive healthcare for children aged 1–3 years.

To enable adequate, rapid, and feasible lifestyle evaluation in young children, to provide parents and youth healthcare professionals (YHCP) guidance in discussing and improving children’s lifestyle behaviour, and ultimately to prevent children from adverse lifestyle-related health consequences, we developed a screening tool called “Features of Lifestyle in Young Kids” (FLY-Kids). The aim of this paper is to (1) describe the development of FLY-Kids and (2) report on its usability, feasibility, and preliminary effect based on the evaluation study.

## Methods

**Fig. 1:**
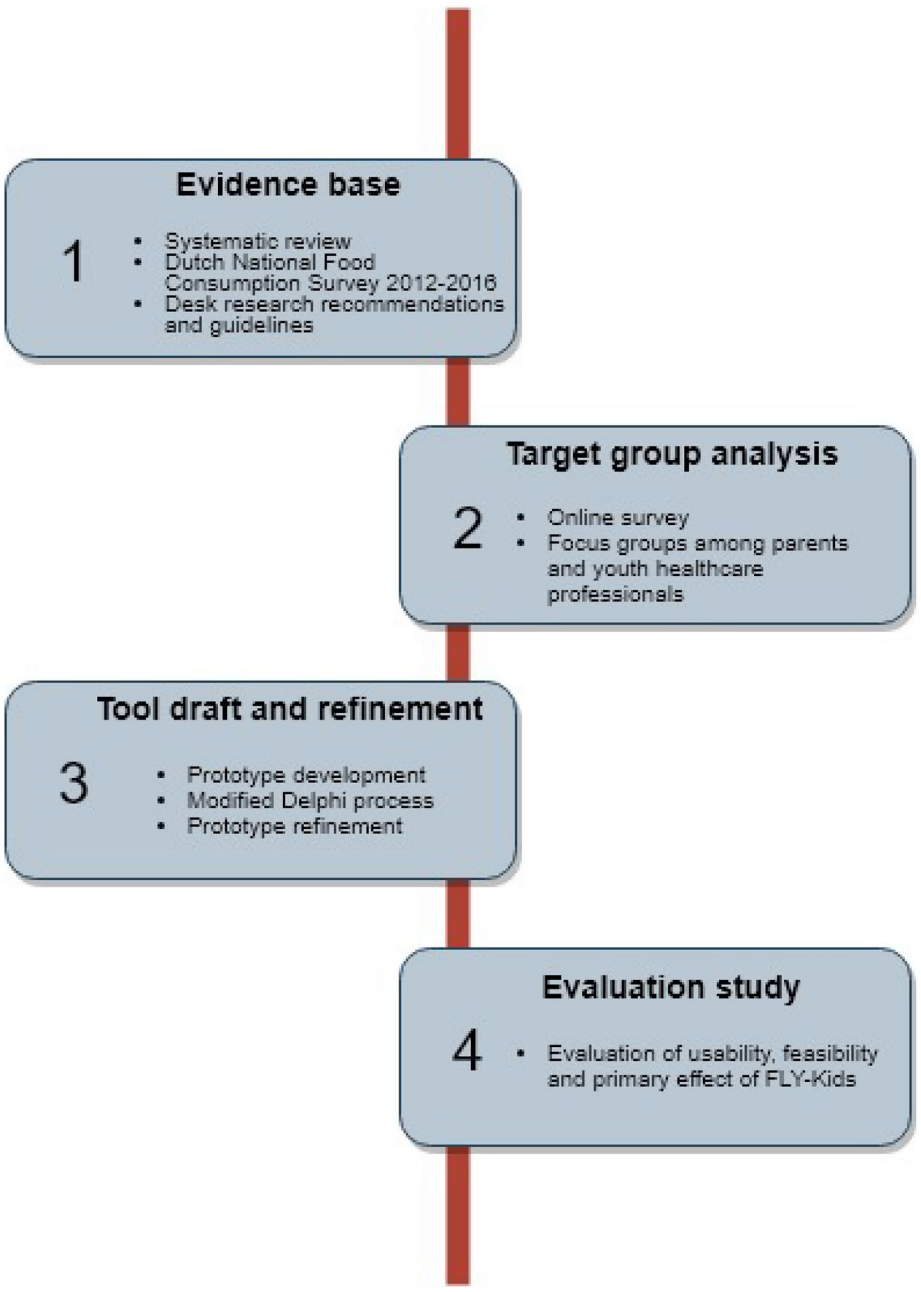
Overview of the development and evaluation process of FLY-Kids

The outline of the development and evaluation process of FLY-Kids is demonstrated in Fig. 1. A detailed description of the development process of FLY-Kids is provided in Online Resource 1.

FLY-Kids is a 10-item parent-administered lifestyle screening tool for children aged 1–3 years (Online Resource 2). The first item determines parental satisfaction with their child’s lifestyle; the other items are divided into four themes and consist of questions that are evaluated against age-specific recommendations: healthy food intake (vegetables and fruits), unhealthy food intake (sugar-sweetened beverages and snacks), eating habits (mealtime practice and food parenting practice), and other lifestyle habits (physical activity, screen time, and sleep). Parents grade their satisfaction on a scale from 1 (very unsatisfied) to 10 (very satisfied). The other questions comprise three or four response options. After completion, these multiple choice items are scored “green”, “orange”, or “red”, with an additional “yellow” in case of four response options, indicating the extent to which the recommendation is met [17, 18]. Since the recommendations for screen time and sleep vary slightly by age, there are three FLY-Kids versions for ages 1, 2, and 3 years, respectively (Online Resource 2). FLY-Kids is intended to be completed prior to a youth healthcare visit and provides healthcare professionals with a dashboard showing which lifestyle aspects may require attention. Healthcare professionals can use this dashboard and enclosed courses of action (potential underlying reasons to explore further, as well as advice and information resources for parents) to enter into dialogue with parents and support them in improving the lifestyle of their child.

### Evaluation study of FLY-Kids

#### Study design and population

Between June and November 2022, FLY-Kids was evaluated at four youth healthcare centres in different municipalities in the Netherlands (Goes, Utrecht, Hardenberg, Almere). These centres were recruited by advertising in the Dutch Knowledge Centre for youth health newsletter and direct communication. We invited parents and their children aged 1–3 years attending a regular youth healthcare appointment. Exclusion criteria were (1) parents not having sufficient command of the Dutch language to complete the tool, (2) parents or children considered not eligible according to the YHCP (e.g. due to psychosocial problems within the family, psychomotor retardation, or a specific diet), or (3) no time to fill out the questionnaire before the appointment. The consulting YHCP (physicians and nurses) were included as a separate participant group.

#### Data collection

A detailed description of the data collection of the evaluation study is described in Online Resource 3. In brief, parents were invited to participate by a researcher in the waiting room after their child’s anthropometric measurements were taken. Parents who verbally agreed to participate provided written informed consent and completed a paper version of FLY-Kids. The researcher passed the scored dashboard on to the YHCP. Parents and YHCP discussed the dashboard during the consultation and advice, and more information was provided accordingly. Afterwards, parents filled out a short questionnaire on background characteristics, and both parents and YHCP completed an evaluation form regarding FLY-Kids’ usability and feasibility on a scale of 1 (strongly disagree) to 5 (strongly agree), with the option to provide additional open text input.

### Statistical analyses

Characteristics of participating children and parents were described in means with standard deviation (SD) and percentages. The normal distribution of continuous variables was tested using histograms and Kolmogorov-Smirnov tests for normality. The mean value of the FLY-Kids item on parental satisfaction was calculated. For the other FLY-Kids items, the proportion of parents who had given the “green”, “yellow”, “orange”, or “red” response option were expressed. Associations of scores on FLY-Kids with parental satisfaction, age of the child, weight SD score, and items discussed during the consultation were examined with Pearson’s correlation coefficient. Likert scale responses on the usability and feasibility questions of parents and YHCP were summarised by means of descriptive statistics. Open text answers were organised by theme and analysed accordingly. SPSS software (IBM SPSS Statistics for Windows, Version 28.0.1.0 NY: IBM Corp.) was used for all quantitative analyses.

## Results

### Sample characteristics

Of the 210 invited parents, 208 agreed to participate. After excluding incomplete (not completed at all, *n* = 1; missing on satisfaction item, *n* = 1), younger age (< 1 year, *n* = 1), and unconsented questionnaires (*n* = 4), 201 were included for analysis. The sample of children comprised 105 1-year-olds (52%), 73 2-year-olds (36%), and 23 3-year-olds (11%), of which 49% were boys (Table 1). Mean SD scores for weight-for-height and height-for-age for all enrolled children were − 0.08 (SD 1.08) and 0.18 (1.26), respectively. As for weight classification, 7% of children were underweight, 81% had a normal weight, 11% had overweight, and 2% were affected with obesity [19, 20]. Participating parents were mostly mothers (75%) and had a mean age of 34.9 y (SD 6.1). In addition, the majority of them were born in the Netherlands (82%) and had attained a high level of education (62%). The evaluation study involved 18 YHCP, of whom 15 completed the evaluation form. Among the latter were 6 (40%) physicians and 9 (60%) nurses.

**Table 1:** Characteristics of children and parents in the evaluation study of FLY-Kids

	All	1 year	2 and 3 years
Number of participants	201	105	96
**Child characteristics**			
Age (months)	22 (8.5)	15 (2.8)	30 (6.1)
Sex, m:v (%)	49:51	49:51	49:51
Weight-for-height SD score	− 0.08 (1.08)	− 0.09 (1.09)	− 0.08 (1.07)
Height-for-age SD score	0.18 (1.26)	0.19 (1.36)	0.18 (1.15)
Weight classification (%)			
Underweight	7	7	7
Normal weight	81	81	80
Overweight	11	12	11
Obesity	2	1	2
**Parent characteristics**			
Relationship with child (%)			
Mother	75	78	71
Father	23	19	27
Other	2	3	2
Age (years)	34.9 (6.1)	34.3 (6.4)	35.6 (5.8)
Country of birth (%)			
The Netherlands	82	82	81
Other Western country	4	6	3
Non-Western country	14	12	16
Education level (%)			
Low	10	7	14
Middle	28	34	22
High	62	59	64

Values are means with standard deviations or percentages

### FLY-Kids scores

**Fig. 2:**
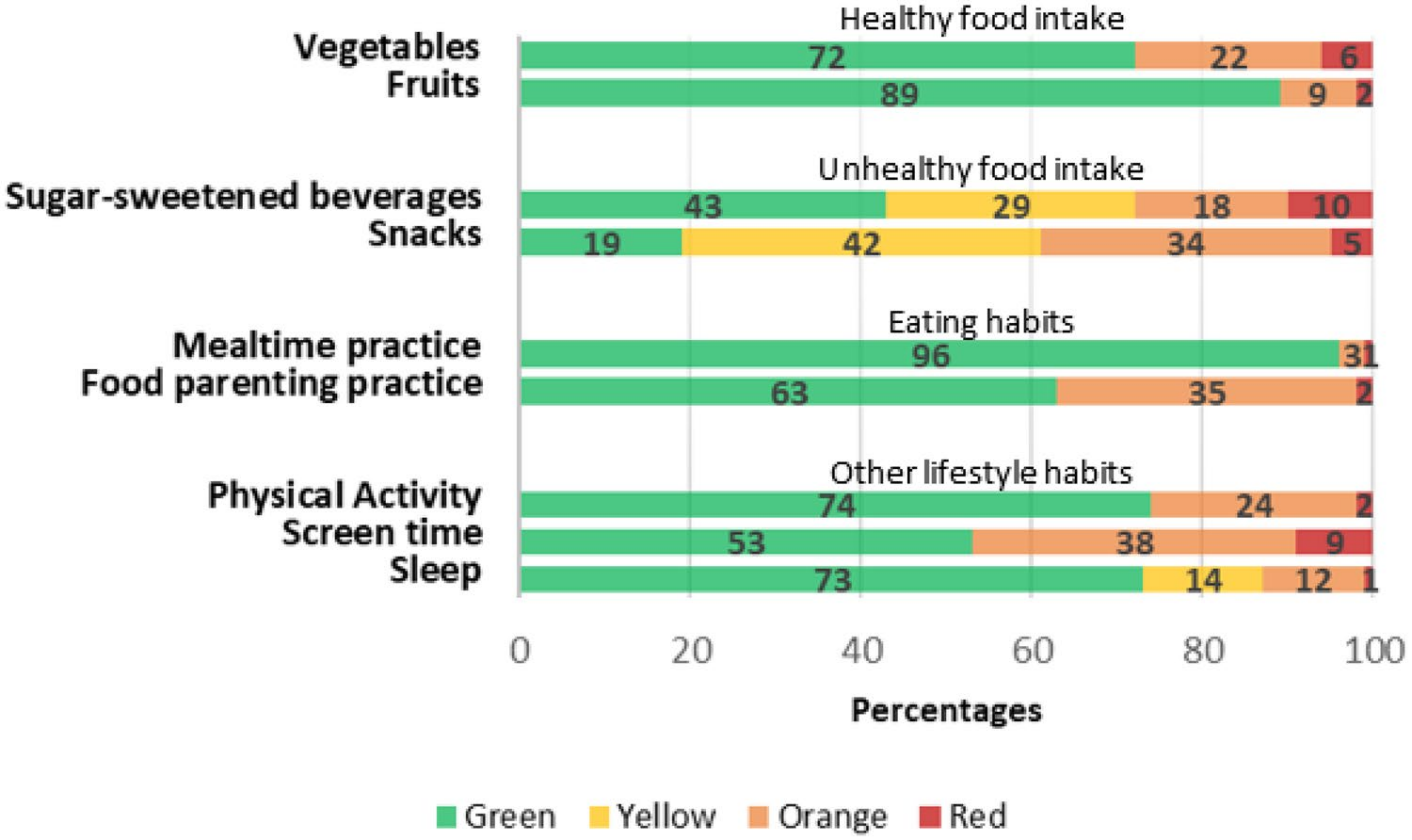
FLY-Kids scores (as compared to national recommendations)

Parents reported a mean satisfaction level of 8.4 (SD 1.0, range 6–10) with regard to their child’s overall lifestyle. The scores on the other FLY-Kids items are demonstrated in Fig. 2. A proportion of 72% of children scored “green” on the item vegetables, meaning they complied with the age-specific recommendation. For fruit, this was 89%, for sugar-sweetened beverages 43%, and for snacks 19%. Parents reported the most favourable response option in 96% and 63% of cases on mealtime practice and food parenting practice items, respectively. Regarding physical activity, screen time, and sleep, parents indicated that their child met the recommendation in 74%, 53%, and 73% of cases, respectively. A total of 6 children (3.0%) scored “green” on all items. On average, children scored 3.2 items (SD 1.6, range 0–9, median 3) that did not meet the recommendation (indicated as “yellow”, “orange”, or “red”) and 2.3 items (SD 1.7, range 0–8, median 2) that required further exploration according to the work instruction (indicated as “orange” or “red”).

Parents who scored high on the satisfaction scale indicated fewer items not meeting the recommendation (*r* =  − 0.32, *p* < 0.001). The age of the children was also associated with the number of items not meeting the recommendation (*r* = 0.30, *p* < 0.001), with younger children having fewer unfavourable scored items. We found no association between the number of items that did not meet the recommendation and the weight-for-height SD score of the children (*r* =  − 0.03, *p* = 0.72).

### Usability and feasibility of FLY-Kids

#### Parents

As to usability of FLY-Kids, parents rated the completion ease with a mean of 4.8 (SD 0.4, range 3–5) (Table 2). The mean rating on clarity of the questions was 4.8 (SD 0.4, range 3–5). Helpfulness of FLY-Kids in the conversation with the YHCP and helpfulness of FLY-Kids-related tips and advice received were scored with an average of 4.4 (SD 0.8, range 2–5) and 4.5 (SD 0.7, range 2–5), respectively. Regarding feasibility, parents rated the completion time with a mean of 4.9 (SD 0.4, range 2–5) and willingness to complete FLY-Kids regularly with a mean of 4.0 (SD 1.1, range 1–5). A total of 36 parents provided an additional open text response. The themes “overall experience”, “snacks”, “digitalisation”, “free text option”, “language”, and “miscellaneous” were used to categorise these responses, which mainly concerned tips for further implementation.

**Table 2:** Usability and feasibility of FLY-Kids according to parents and YHCP

**Parents**			
**Usability**		**Feasibility**	
**Item**	**Rating, mean (SD, range**^**a**^**)**	**Item**	**Rating, mean (SD, range**^**a**^**)**
Completion ease	4.8 (0.4, 3–5)	Completion duration	4.9 (0.4, 2–5)
Clarity of questions	4.8 (0.4, 3–5)	Willingness regular completion	4.0 (1.1, 1–5)
Helpfulness in conversation	4.4 (0.8, 2–5)		
Helpfulness of tips and advice	4.5 (0.7, 2–5)		

**YHCP**			
**Usability**		**Feasibility**	
**Item**	**Rating, mean (SD, range**^**a**^**)**	**Item**	**Rating, mean (SD, range**^**a**^**)**
User-friendliness	4.6 (0.7, 3–5)	Practicality during consultation	4.1 (0.9, 3–5)
Clarity of utilisation	4.8 (0.4, 4–5)	Compatibility with working practice	4.1 (0.7, 3–5)
Helpfulness of dashboard	4.5 (0.6, 3–5)	Possibility integration within consultation time	3.7 (1.1, 1–5)
Helpfulness in conversation	4.5 (0.6, 3–5)	Satisfaction of parents	4.1 (0.8, 3–5)
		Workability of courses of action	4.3 (0.8, 3–5)

^a^Potential range was 1–5

#### YHCP

Concerning usability of FLY-Kids, YHCP scored the overall user-friendliness with an average of 4.6 (SD 0.7, range 3–5) and the clarity of how to use the screening tool with a mean of 4.8 (SD 0.4, range 4–5) (Table 2). Helpfulness of the dashboard in providing an overview of the child’s lifestyle and helpfulness of FLY-Kids in the conversation were rated with mean values of 4.5 (SD 0.6, range 3–5) and 4.5 (SD 0.6, range 3–5), respectively. As to feasibility, practicality of using FLY-Kids during the consultation scored a mean of 4.1 (SD 0.9, range 3–5). YHCP rated the compatibility with regular working practice and possibility of integration within the consultation time constraints with means of 4.1 (SD 0.7, range 3–5) and 3.7 (SD 1.1, range 1–5), respectively. In addition, they scored the satisfaction of parents when using FLY-Kids with a mean of 4.1 (SD 0.8, range 3–5) and the workability of the courses of action with a mean of 4.3 (SD 0.8, range 3–5). Open text responses by YHCP were classified in the themes “digitalisation”, “nuance within responses”, and “concerns for implementation”.

### Preliminary effects of FLY-Kids

A majority of parents (96%) reported having discussed their child’s lifestyle with the YHCP during the consultation. The YHCP reported an average of 2.9 FLY-Kids items (SD 2.4, range 0–9, median 2) discussed. The number of items scored “orange” or “red” was associated with the number of items discussed during the consultation (*r* = 0.47, *p* < 0.001).

## Discussion

This paper describes the development and first evaluation study of FLY-Kids, a lifestyle screening tool for children aged 1–3 years. Following the development process, we showed that most parents were willing to complete FLY-Kids and considered it helpful and easy to use. YHCP confirmed this usefulness and discussed with parents items marked as requiring further exploration.

Parents scored an average of 3.2 (out of 9) unfavourable lifestyle behaviours in their children, and only 3.0% of children complied with all recommendations. These findings suggest that FLY-Kids is able to identify unhealthy behaviour and that young children may benefit from lifestyle screening through FLY-Kids, via targeted advice for lifestyle improvement by their parents. Most unfavourable lifestyle behaviours were reported in unhealthy food intake (sugar-sweetened beverages and snacks) and electronic screen time behaviour. These results are in accordance with the previous population studies that demonstrated that young children regularly consume sugar-sweetened beverages and snacks that are high in salt, sugar, and saturated fats [21]. Concerning usage of electronic screens, our results also concur with the former studies that concluded that a major proportion of young children does not meet screen time guidelines [22].

Interestingly, parents who scored high on the satisfaction scale scored more items meeting the recommendation. It cannot be inferred from our results whether following more recommendations increased parents’ satisfaction with their child’s lifestyle or the other way around. However, in line with the potential benefits of motivational interviewing for lifestyle behaviour change, we consider determining parental satisfaction a relevant component of FLY-Kids [23].

Overall, we discovered end-user support for the use of FLY-Kids within youth healthcare, a crucial condition for successful implementation. Regarding the usability, parents and YHCP reported that the screening tool was simple and easy to use. Furthermore, we observed that both parents and YHCP regarded FLY-Kids to be helpful in the conversation. As this user experience matches the goal of FLY-Kids, i.e. to screen young children’s lifestyle in order to support a conversation about lifestyle between parents and YHCP, this is an encouraging finding. Moreover, YHCP felt they were given a good overview of children’s lifestyle and parents valued the tips and advice they received. FLY-Kids’ feasibility for use in youth healthcare was also rated fairly high, albeit lower than its usability. For YHCP, this was mainly due to the limited consultation time. As also mentioned by several parents, digitalisation of FLY-Kids may increase its usability. In addition, a digital version may enhance integration with the electronic health record, saving time and increasing feasibility, and enable longitudinal measurements.

In 96% of cases, parents reported they had discussed their child’s lifestyle with the YHCP during the consultation. While parents scored an average of 2.3 items that needed further exploration or discussion according to the work instruction (i.e. items scored orange or red), an average of 2.9 FLY-Kids items were actually discussed during the consultation. Furthermore, we found a strong association between the number of items requiring further exploration and the number of items discussed. These results suggest that FLY-Kids promotes a conversation about lifestyle that is broader than the aspects that may require attention.

However, the crucial step in improving children’s lifestyle lies in incorporating the information and advice and actual lifestyle behaviour change. Ultimately, this would lead to positive health outcomes, such as maintaining a healthy weight. In the evaluation study, we could not determine an association between the number of items that did not meet the recommendation and the weight-to-height SD score of the children. Such outcome validation would provide evidence that FLY-Kids is a valuable tool in identifying children at the highest risk for lifestyle-related health problems. Longitudinal research is needed to determine whether the use of FLY-Kids contributes to positive lifestyle behaviour change and associated health benefits.

### Strengths and limitations

FLY-Kids was created through an extensive development process. By first evaluating parental satisfaction and provision of specific courses of action, YHCP are assisted in engaging into an open dialogue with the parent and tailoring advice to fit the family concerned. We consider these features to be the major strengths of the tool. The high response rate of the evaluation study suggests that FLY-Kids is undemanding and can be used in preventive healthcare settings with limited consultation time. In addition, as the aim of FLY-Kids is general and the items are relevant to all young children, we consider the tool to be generalisable to other countries.

As discussing lifestyle is incorporated in standard care and we did not include a control group, it could not be inferred from our findings whether FLY-Kids ensures more frequent lifestyle dialogues. In addition, the presence of the researcher may have resulted in more awareness and prompts to talk about lifestyle and more socially desirable responses. The latter is also a potentially negative feature of self-reporting in general. Although the evaluation study was carried out in areas with varying degrees of urbanisation, only a small percentage of parents had a low education level and/or migration background. Given that these families may have higher odds for having an unhealthy lifestyle, we consider this another study limitation [24, 25]. Lastly, some locations also offered telephone instead of in-person consultations to 2- and 3-year-olds, leading to a lower number of evaluated children within these age groups.

## Conclusions

FLY-Kids is a screening tool designed to rapidly evaluate multiple dimensions of lifestyle in children aged 1–3 years. It allows YHCP to use a dashboard with outcomes as a conversation tool to provide parents with tailored support towards behaviour change. FLY-Kids’ usability and feasibility were highly rated by parents and YHCP. In addition, during the preventive healthcare consultation, parents and YHCP were able to discuss lifestyle items identified by FLY-Kids as requiring attention. Longitudinal research is needed to determine whether the use of FLY-Kids contributes to positive lifestyle behaviour change and associated health benefits.

### Supplementary Information

Below is the link to the electronic supplementary material.

Online Resource 1 (PDF 416 KB)

Online Resource 2 (PDF 555 KB)

Online Resource 3 (PDF 401 KB)

## Data Availability

The data used in this study are available upon request from the corresponding author.

## Abbreviations

FLY-Kids: Features of Lifestyle in Young Kids
YHCP: Youth healthcare professionals
