# Supplementary material for: Development and evaluation study of FLY-Kids: a new lifestyle screening tool for young children
Source: Eur J Pediatr. 2023 Aug 15;182(10):4749–57. doi: 10.1007/s00431-023-05126-6 (PMC10587277; doi:10.1007/s00431-023-05126-6)
Supplement: Supplementary file 1 — Online Resource 1 (PDF 416 KB) [file 431_2023_5126_MOESM1_ESM.pdf]

FLY-Kids **Please tick the box of your choice for each question**

**1. How satisfied are you with your child's lifestyle (diet, physical activity, screen time, sleep)?**<br>Circle a number between 1 and 10	Satisfaction<br>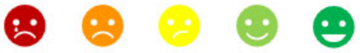<br>1 2 3 4 5 6 7 8 9 10
**2. How many vegetables does your child eat per day?**<br>Consider all vegetables your child consumes, including, for example, cucumber in between meals.<br>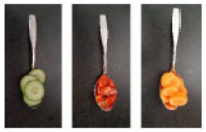<br>Examples of 1 serving spoon of vegetables	☐ Less than half a serving spoon a day<br>☐ Half to 1 serving spoon a day<br>☐ 1 serving spoon or more a day
**3. How many days a week does your child eat fruit?**<br>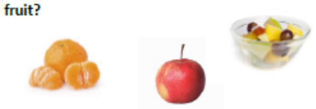	☐ Less than 4 days a week<br>☐ 4 to 6 days a week<br>☐ Every day
**4. How many sugar-sweetened beverages does your child drink per day?**<br>Consider, for example, soft drinks, fruit juice, thick juice, lemonade, and milk drinks with sugar, such as chocolate milk and yoghurt drink.<br>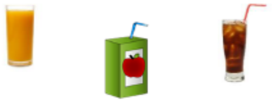	☐ None<br>☐ Less than 1 glass or juice box a day<br>☐ 1 glass or juice box a day<br>☐ 2 glasses or juice boxes or more a day
**5. How many snacks does your child eat per day?**<br>Consider, for example, cookies, candy, crisps, and cake.<br>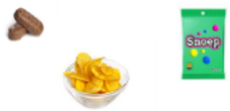 	☐ None<br>☐ Less than 1 snack a day<br>☐ 1 snack a day<br>☐ 2 snacks or more a day

**6. How often does your child eat his/her meals at the dining table?**<br>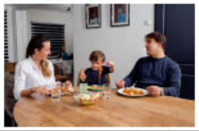	☐ Almost never<br>☐ Occasionally<br>☐ Almost always
**7. How often do you give your child something to eat to comfort or reward him/her?**<br>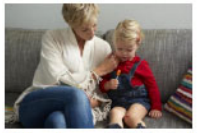	☐ Almost never<br>☐ Occasionally<br>☐ Almost always
**8. How much time is your child physically active per day?**<br>Consider, for example, active (outdoor)playing, cycling, crawling, playing with a ball, moving to music, running and jumping.<br>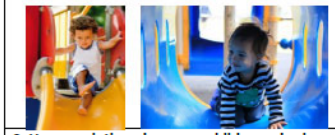	☐ Less than 1.5 hours a day<br>☐ 1.5 to 3 hours a day<br>☐ 3 hours or more a day
**9. How much time does your child spend using electronic screens per day?**<br>Consider, for example, TV, computer, mobile phone and tablet.<br>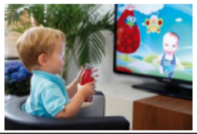	☐ 0 hours a day<br>☐ Less than 1 hour a day<br>☐ More than 1 hour a day
**10. How much time does your child sleep per 24 hours?**<br>Also include naps during daytime.<br>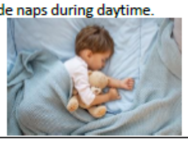	☐ Less than 9 hours per 24 hours<br>☐ 9 to 11 hours per 24 hours<br>☐ 11 to 14 hours per 24 hours<br>☐ More than 14 hours per 24 hours

Dashboard:

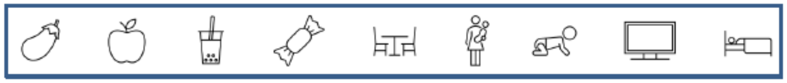

Supplementary Figure 1: FLY-Kids tool for children aged 1 year (translated and back translated from and to Dutch)

Note: Response options for **screen time** for children aged 2 and 3 years are: 0 to 1 hour a day, 1 to 2 hours a day, and 2 hours or more a day. Response options for **sleep** for children aged 3 years are: less than 8 hours per 24 hours, 8 to 10 hours per 24 hours, 10 to 13 hours per 24 hours, and more than 13 hours per 24 hours.
