# Supplementary material for: Development and evaluation study of FLY-Kids: a new lifestyle screening tool for young children
Source: Eur J Pediatr. 2023 Aug 15;182(10):4749–57. doi: 10.1007/s00431-023-05126-6 (PMC10587277; doi:10.1007/s00431-023-05126-6)
Supplement: Supplementary file 2 — Online Resource 2 (PDF 555 KB) [file 431_2023_5126_MOESM2_ESM.pdf]

Supplementary Text File 1: Detailed description of the development of the lifestyle screening tool FLY-Kids

A consortium of directly involved parties was established at the start. Throughout the project, this consortium met every few months for updates and discussion. In addition, once a year, a larger group of experts united in the Dutch "Platform Healthy Nutrition 0-4 years" [1] held a meeting about the project.

Phase 1: Evidence base

In phase 1, we established the scientific background of the screening tool under development.

Systematic review: A systematic review was conducted to identify existing lifestyle screening tools for children in the community setting and to gain insight into their features of design, psychometric properties and implementation [2].

Dutch National Food Consumption Survey 2012-2016: The Dutch National Food Consumption Survey 2012-2016 was also used in the development of FLY-Kids. Observed dietary intakes and derived lifestyle clusters of children aged 1-3 years provided information on potential nutritional challenges and underlying patterns, respectively [3, 4].

Desk research recommendations and guidelines: In addition, we performed desk research into age-specific lifestyle recommendations and guidelines for healthcare professionals and associated advice and information resources for parents to outline lifestyle topics with available courses of action [5-7].

Phase 2: Target group analysis

Phase 2 involved target group analysis (results not presented).

Online survey and focus groups: We conducted an online survey among parents of young children and focus group discussions among parents (N=25) and YHCP (N=25) to identify the needs and wishes for the lifestyle screening tool under development. Using a topic guide, we consecutively addressed: 1) the role of youth healthcare in young children's lifestyle, and 2) the requirements for the lifestyle screening tool under development.

Phase 3: Tool draft and refinement

Prototype development: A prototype of FLY-Kids was developed in phase 3. To support YHCP in aligning with parents' perceptions during the healthcare visit, the first item was constructed to address the parental perspective on their child's lifestyle. For the other items in the tool, we restricted the list of potential topics emerging from phases 1 and 2 to topics concerning modifiable lifestyle behaviour of the child. Topics selected for the prototype had to be associated with health outcomes in children. Moreover, age-specific recommendations and courses of action had to be available in the case of unfavourable behaviour. Items were formulated at Dutch language level B1. For each lifestyle item in the prototype, potential courses of action were compiled based on the desk research.

Modified Delphi process: Then, using a modified Delphi process, the content of the 10-item prototype and courses of action were evaluated by a group of experts (paediatricians, youth healthcare physicians, dietitians, nutrition scientists, and policy officers) in two online survey rounds [8]. In round 1, participants were to express their opinion on the FLY-Kids items (questions and response options), choosing from: "Fine, keep in present form", "Small modification, namely:…", "Other question with this

topic, namely:…". A free text field was included for suggestions and recommendations. In addition, participants were asked to add to the lists of courses of action. Round 1 was completed by 30 participants, with agreement ranging from 23-80%. Revision of the prototype was based on agreement and free text input. In round 2, participants had to indicate whether they agreed with the modified items and lists of courses of action ("Yes, I agree", "No, I do not agree"). Again a free text field was included. A total of 25 participants completed round 2. Agreement for the FLY-kids items ranged from 76-100%. For the courses of action, an agreement between 88-100% was reached.
Prototype refinement: A meeting with the Platform Healthy Nutrition 0-4 years was organised to discuss final modifications and to agree upon the final content of FLY-Kids. Finally, FLY-Kids was provided with supporting images to assist parents in completing the screening tool (Online Resource 1).

References

1. Platform Gezonde Voeding 0-4 jaar [Available from: https://www.platformkindervoeding.nl/].
2. Krijger A., Ter Borg S., Elstgeest L., van Rossum C., Verkaik-Kloosterman J., Steenbergen E., et al. Lifestyle screening tools for children in the community setting: A systematic review. Nutrients. 2022;14(14).
3. Steenbergen E., Krijger A., Verkaik-Kloosterman J., Elstgeest L. E. M., Ter Borg S., Joosten K. F. M., et al. Evaluation of nutrient intake and food consumption among Dutch toddlers. Nutrients. 2021;13(5).
4. Krijger A., Steenbergen E., Schiphof-Godart L., van Rossum C., Verkaik-Kloosterman J., Elstgeest L., et al. Clusters of lifestyle behaviours and their associations with socio-demographic characteristics in Dutch toddlers. Eur J Nutr. 2022.
5. The Netherlands Nutrition Centre. The Hague, the Netherlands, https://www.voedingscentrum.nl.
6. Dutch Knowledge Centre for Youth Health. Utrecht, the Netherlands, www.ncj.nl
7. Dutch Society of Pediatrics. Utrecht, the Netherlands, www.nvk.nl.
8. Barrett D., Heale R. What are Delphi studies? Evid Based Nurs. 2020;23(3):68-9.
