# Supplementary material for: Development and evaluation study of FLY-Kids: a new lifestyle screening tool for young children
Source: Eur J Pediatr. 2023 Aug 15;182(10):4749–57. doi: 10.1007/s00431-023-05126-6 (PMC10587277; doi:10.1007/s00431-023-05126-6)
Supplement: Supplementary file 3 — Online Resource 3 (PDF 401 KB) [file 431_2023_5126_MOESM3_ESM.pdf]

Supplementary Text File 2: Detailed description of the data collection within the evaluation study of FLY-Kids

Upon arrival in the waiting room and as part of standard care, anthropometric measurements of the children were performed according to standardised protocols. A doctor's assistant or trained researcher weighed children (wearing no or light underclothes) to the nearest 100 grams using a calibrated mechanical or digital scale. An infantometer and stadiometer were used to measure the height of children below and above two years, respectively, to the nearest 1 millimetre.

Soon after, parents were informed about the aim of the study and invited to participate by a researcher. Parents who had verbally agreed to participate provided written informed consent, completed the paper form of FLY-Kids, and returned all paper forms to the researcher. Only parents who provided written informed consent were included in the study. The researcher scored the FLY-Kids items and passed the form on to the youth healthcare professional (YHCP).

The YHCP then used FLY-Kids during the consultation to initiate the conversation about lifestyle. More specifically, the parents' satisfaction with their child's lifestyle and the parents' questions were addressed, and items scored "orange" or "red" were further explored. More information and advice were given accordingly. YHCP marked the discussed FLY-Kids items on the form.

After the consultation, parents reported some characteristics about themselves and responded to statements about the usability and feasibility of FLY-Kids on a scale of 1 (strongly disagree) to 5 (strongly agree) and could give additional written feedback. The YHCP evaluated the usability and feasibility of FLY-Kids, also on a scale of 1 (strongly disagree) to 5 (strongly agree) via an online form following the evaluation period at the healthcare centre where they were employed.
